# Binaural Processing Deficits Due to Synaptopathy and Myelin Defects

**DOI:** 10.3389/fncir.2022.856926

**Published:** 2022-04-14

**Authors:** Maral Budak, Michael T. Roberts, Karl Grosh, Gabriel Corfas, Victoria Booth, Michal Zochowski

**Affiliations:** ^1^Biophysics Program, University of Michigan, Ann Arbor, MI, United States; ^2^Department of Microbiology and Immunology, University of Michigan Medical School, Ann Arbor, MI, United States; ^3^Department of Otolaryngology Head and Neck Surgery, University of Michigan, Ann Arbor, MI, United States; ^4^Kresge Hearing Research Institute, University of Michigan, Ann Arbor, MI, United States; ^5^Department of Mechanical Engineering, University of Michigan, Ann Arbor, MI, United States; ^6^Department of Biomedical Engineering, University of Michigan, Ann Arbor, MI, United States; ^7^Department of Mathematics and Anesthesiology, University of Michigan, Ann Arbor, MI, United States; ^8^Department of Physics, University of Michigan, Ann Arbor, MI, United States

**Keywords:** hidden hearing loss (HHL), synaptopathy, myelin abnormalities, binaural processing deficits, medial superior olive, computational model

## Abstract

Hidden hearing loss (HHL) is a deficit in auditory perception and speech intelligibility that occurs despite normal audiometric thresholds and results from noise exposure, aging, or myelin defects. While mechanisms causing perceptual deficits in HHL patients are still unknown, results from animal models indicate a role for peripheral auditory neuropathies in HHL. In humans, sound localization is particularly important for comprehending speech, especially in noisy environments, and its disruption may contribute to HHL. In this study, we hypothesized that neuropathies of cochlear spiral ganglion neurons (SGNs) that are observed in animal models of HHL disrupt the activity of neurons in the medial superior olive (MSO), a nucleus in the brainstem responsible for locating low-frequency sound in the horizontal plane using binaural temporal cues, leading to sound localization deficits. To test our hypothesis, we constructed a network model of the auditory processing system that simulates peripheral responses to sound stimuli and propagation of responses via SGNs to cochlear nuclei and MSO populations. To simulate peripheral auditory neuropathies, we used a previously developed biophysical SGN model with myelin defects at SGN heminodes (myelinopathy) and with loss of inner hair cell-SGN synapses (synaptopathy). Model results indicate that myelinopathy and synaptopathy in SGNs give rise to decreased interaural time difference (ITD) sensitivity of MSO cells, suggesting a possible mechanism for perceptual deficits in HHL patients. This model may be useful to understand downstream impacts of SGN-mediated disruptions on auditory processing and to eventually discover possible treatments for various mechanisms of HHL.

## Introduction

**FIGURE 1:**
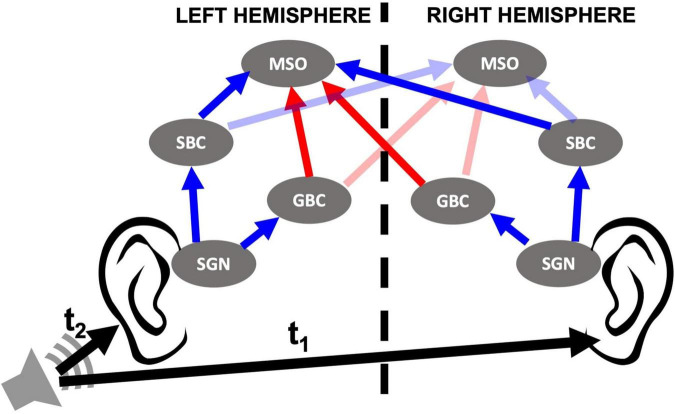
Cochlear nucleus circuit model containing spiral ganglion neurons (SGNs), spherical bushy cells (SBCs), globular bushy cells (GBCs), and medial superior olives (MSOs) of both sides. Sound stimuli trigger release events at inner hair cell (IHC)-SGN synapses that stimulate SGN fibers as described in Budak et al. (2021). SGNs then relay the signal to the ipsilateral SBCs and GBCs in the cochlear nucleus. MSO cells in the brainstem receive excitatory (blue arrows) and inhibitory (red arrows) inputs from SBCs and GBCs, respectively, from both sides. LNTB and MNTB that relay input from ipsi- and contralateral GBCs, respectively, are omitted from the model and therefore not shown in the circuit. The horizontal location of the sound source is defined by interaural time difference (ITD), the difference in the arrival time of the sound to both ears (t1–t2). ITD > 0 when sound comes from left and ITD < 0 when sound comes from right.

The ability to determine the location of the source of a sound is critical for all animals. They can more easily find prey, escape from predators and survive other dangers in nature thanks to their sound localization skills. Humans, as well, benefit from this ability to assess their safety and to distinguish speech when competing sounds are present. Unlike visual and somatosensory systems, the auditory system does not map the spatial origin of stimuli onto the sensory epithelium. Instead, the brain uses temporal, spectral, and intensity cues to determine the location of the source of sounds in three-dimensional space (Middlebrooks, 2015). Locating a sound in the horizontal plane requires precise temporal and intensity information coming from both ears. Integration of binaural information in humans takes place in the superior olivary complex (SOC) located in the brainstem, specifically in the medial superior olive (MSO), where azimuthal sound localization occurs. MSO cells receive binaural excitatory inputs from spherical bushy cells (SBCs) and binaural inhibitory inputs driven by globular bushy cells (GBCs), which act via relay nuclei, the medial and lateral nuclei of the trapezoid body (MNTB and LNTB) (Scott et al., 2005). SBCs and GBCs are located in the cochlear nucleus, the first relay point for signals from the periphery to the central auditory system. Multiple spiral ganglion neurons (SGNs) project from the cochlea to SBCs and GBCs and anatomical studies show that SBCs typically receive input from 2 to 4 SGNs while GBCs receive input from 9 to 69 SGNs (Sento and Ryugo, 1989; Liberman, 1991; Ryugo and Sento, 1991; Spirou et al., 2005). Integration of input from multiple SGNs, along with specializations in synaptic and intrinsic physiology, enable SBCs and GBCs to respond to sound with more precise phase-locking than the SGN fibers, therefore transmitting precise timing information to MSO cells (Joris et al., 1994). A reduced representation of this circuitry is shown in Figure 1.

Humans can resolve interaural time differences (ITDs), the difference in the arrival time of a sound to each ear, as short as 10 μs, and can locate sound sources as precisely as a few degrees of azimuth (Kandel et al., 2000). MSO cells participate in sound localization by acting as coincidence detectors with their firing activity showing sensitivity to ITD levels (Yin and Chan, 1990). Specifically, MSO cells, on one side of the brain, do not fire unless they are excited by contra- and ipsilateral SBCs within a short time window. For binaural excitatory inputs to coincide at an MSO on one side of the brain, the sound needs to arrive to the contralateral ear first. In this way, the internal delay caused by the signal traveling from the contralateral ear is compensated for. As a result, the ITDs inducing the highest activity in the MSO on one side of the brain, also called the best ITDs, correspond to slightly contralateral-leading sound source locations. This gives rise to different MSO responses in each side to the same sound, and the horizontal location of the sound is encoded in the brain by the difference in firing rates between the MSOs in each side (Harper and McAlpine, 2004).

The precise timing of binaural signals is essential to detect the horizontal direction of the sound source. Therefore, the disruption of signaling along peripheral auditory circuits would significantly impair sound localization ability in humans. Many behavioral and electrophysiological studies suggest that humans with normal audiometric thresholds can have problems with encoding and processing binaural cues, giving rise to speech intelligibility and ITD sensitivity deficits. These deficits occur as a result of noise exposure (Bharadwaj et al., 2015; Bernstein and Trahiotis, 2016; Prendergast et al., 2017), aging (Eddins et al., 2018) or demyelinating diseases (Rance et al., 2012; Furst and Levine, 2015; Choi et al., 2018), and lead to a condition known as hidden hearing loss (HHL). In this study, we hypothesize that these perceptual deficits stem from the lack of coincidence of inputs from SBCs to MSO cells, leading to decreased activity levels of MSO cells and deficits in ITD discrimination. To test this hypothesis, we constructed a network model of binaural auditory processing from the periphery to the MSO. We employed the computational model from Budak et al. (2021) to simulate the response of SGN fibers to sound stimuli under conditions simulating either myelin defects at SGN heminodes or synapse loss at inner hair cell (IHC)-SGN synapses, since animal studies suggested that myelinopathy in the SGN heminodes (Wan and Corfas, 2017) and synaptopathy at IHC-SGN synapses (Kujawa and Liberman, 2009; Furman et al., 2013; Sergeyenko et al., 2013; Viana et al., 2015; Gleich et al., 2016; Valero et al., 2017) result in HHL. We additionally simulated the activity of SBC and GBC populations, and their projections to MSO populations. Model results show decreased firing rates of MSO cells for varying ITDs with increasing degrees of myelinopathy and synaptopathy, indicating decreased MSO activity and ITD sensitivity. This effect on MSO activity is less pronounced in response to sounds waves at resonant frequencies of MSO cells, i.e., the sound frequencies at which MSO cells exhibit the highest activity. These results provide evidence that HHL-associated peripheral neuropathies might lead to lower MSO ITD sensitivity, especially at non-resonant sound frequencies, which potentially causes sound localization problems and speech intelligibility deficits in HHL patients.

## Results

In this study, we aimed to bridge the gap between observed peripheral auditory deficits in animal models of HHL and mechanisms underlying perceptual deficits in patients with HHL. For that purpose, we modeled a mammalian SOC circuit that includes cochlear sound processing and SGN, SBC, GBC, and MSO cell populations (Figure 1), focusing on the crucial role that MSO cells play in sound localization. We simulated circuit responses to binaural sound stimuli at different azimuthal locations under multiple peripheral auditory deficit conditions that have been shown to cause HHL in animal models, i.e., myelinopathy at the SGN axons (Wan and Corfas, 2017) and synaptopathy at IHC-SGN synapses (Kujawa and Liberman, 2009; Furman et al., 2013; Sergeyenko et al., 2013; Viana et al., 2015; Gleich et al., 2016; Valero et al., 2017). Specifically, we analyzed the population firing rates and phase-locking to sound stimuli while simulating varying degrees of myelinopathy at the SGN axons (Wan and Corfas, 2017) and synaptopathy at IHC-SGN synapses (Kujawa and Liberman, 2009; Furman et al., 2013; Sergeyenko et al., 2013; Viana et al., 2015; Gleich et al., 2016; Valero et al., 2017).

We prescribed SGN myelinopathy levels (see also Budak et al., 2021) by varying the length of the initial unmyelinated segment L_*u*_, such that 0% variation represents a homogeneous population of SGN fibers whose L_*u*_ is 10 μm (putative control condition), whereas 100% variation stands for a heterogeneous SGN population where L_*u*_’s vary between 10 and 20 μm.

Experimental results show that the synaptopathy driven by noise-exposure selectively targets IHC-HT SGN synapses (Furman et al., 2013). Here, for generality, we simulated two types of synaptopathies, high threshold (HT) and random synaptopathies, to compare their differential effects on downstream circuit activity. In HT synaptopathy, we removed an increasing fraction of IHC-HT SGN synapses, leaving all low- (LT) and medium-threshold (MT) synapses intact, such that all HT synapses were intact in 0% synaptopathy (putative control condition) and all HT synapses were removed in 100% synaptopathy. In random synaptopathy, the same number of synapses as the corresponding level of HT synaptopathy was removed across all three types of SGN fibers. All synapses were present at 0% synaptopathy (putative control condition) and 1/3rd of the synapses were randomly removed at 100% synaptopathy. In this way, random synaptopathy acts as a control simulation to understand the specific effect of the loss of IHC-HT SGN synapses on downstream processing.

### Effect of Peripheral Neuropathies on Spike Activity and Dynamics in the Superior Olivary Complex Circuit

**FIGURE 2:**
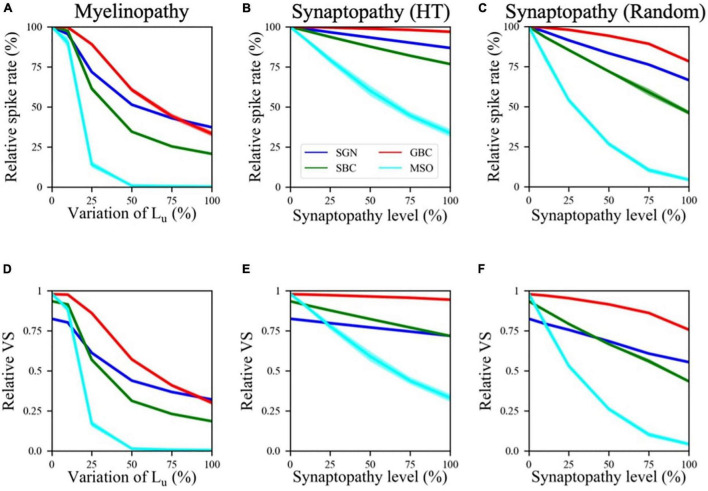
Spike activity and dynamics in all cell populations was disrupted with higher levels of peripheral auditory deficiencies. Relative spike rate **(A–C)** and relative VS **(D–F)** measurements of different cell populations (SGN, SBC, GBC, and MSO) in response to 200 Hz, 50 dB sound stimulus for different peripheral neuropathy conditions: myelinopathy **(A,D)**, synaptopathy at HT **(B,E)** and random synaptopathy **(C,F)**. In myelinopathy, 0% variation of L_u_ represents a circuit with a homogeneous population of SGNs with 10 μm long L_u_, and 100% variation of L_u_ represents a circuit with a heterogeneous SGN population with 10 μm ≤ L_u_ ≤ 20 μm. In both synaptopathy scenarios (HT and random), 0% synaptopathy indicates all IHC-SGN synapses are intact. Synaptopathy level of 100% means all IHC-HT SGN synapses are deficient in HT synaptopathy, whereas the same number of synapses are randomly removed in random synaptopathy (1/3rd of all synapses). In all conditions, increasing the degree of the deficiency (myelinopathy or synaptopathy) decreased relative spike rates and relative vector strength (VS, see “Materials and Methods” section) of all cell types, yet with different slopes for different scenarios. However, in all scenarios, decreases were more pronounced for MSO cells.

First, we explored changes in firing rates of different cell populations as a function of varying myelinopathy and synaptopathy levels (Figure 2). The spike rates of SGN fibers relative to the control (0% L_*u*_ variation or 0% synaptopathy) were significantly decreased with increasing degrees of myelinopathy (Figure 2A) and both HT and random synaptopathies (Figures 2B,C). As predicted, bigger drops in activity were observed in random (Figure 2C) as compared to HT synaptopathy (Figure 2B).

This outcome is in agreement with both experimental studies on animal models (Kujawa and Liberman, 2009; Sergeyenko et al., 2013; Wan and Corfas, 2017) and our computational study of SGN fibers suggesting that myelin defects in SGN fibers or loss of IHC-SGN synapses cause a drop in cumulative SGN activity (Budak et al., 2021). Decreased SGN activity leads to smaller numbers of input spikes to the cochlear nucleus, thus significantly decreasing spike rates of SBCs, GBCs and finally MSO cells as well (Figures 2A–C). Moreover, our results suggest that the activity drop with the increase in any type of peripheral auditory deficit is more significant in SBCs than SGNs and GBCs, and MSO cells show the largest relative decreases in spiking rate (Figures 2A–C). Even though SBCs and GBCs both receive inputs from SGNs, SBCs are affected more than GBCs by both synaptopathy and myelinopathy (Figures 2A–C). This result stems from the fact that GBCs receive a higher number of inputs from SGNs than SBCs, which means that individual GBCs are likely to experience a loss in input closer to the population average loss, whereas SBCs, with only four inputs, can exhibit much larger losses in some cases due to the stochasticity of input loss.

Furthermore, comparing population firing rates in both synapse loss conditions (HT and random synaptopathies) suggests that random synaptopathy has a larger impact on the activities of cochlear nucleus cells, whereas HT synaptopathy barely decreases spike rates of SBCs and GBCs (Figures 2B,C). This difference arises from low activity levels of HT SGN fibers at 50 dB (Winter et al., 1990), resulting in ∼10% decrease in relative spike rates of SGNs when all HT SGN synapses are removed, unlike the case where the same number of synapses are randomly removed, causing ∼35% decrease in SGN activity (Budak et al., 2021). However, relative spike rates of MSO cells still decrease (∼70% decrease in relative spike rate when all HT synapses are removed) more than other cell types in the HT synaptopathy scenario, showing that even negligible drops in SGN inputs may have significant effects on MSO cell firing activity.

Next, we investigated the effect of the SGN neuropathies on phase locking of neuronal firing to the sound wave. The relative vector strength (VS, see “Materials and Methods”) of SGN axons was approximately 0.82 in our control case in response to a 200 Hz, 50 dB sound pulse, while SBCs had a relative VS of 0.93 (Figures 2D–F), indicating increased synchrony compared to the SGN input. The GBC and MSO cell responses were also highly phase-locked to the sound wave, with relative VS approaching to 1.0 (Figures 2D–F). These results agree with experimental observations (Joris et al., 1994; Grothe and Park, 1998) that spike locking to sound increases at SBCs and even more so at GBCs and MSO cells, as compared to SGNs. The relative VS of all cell populations decreased for higher myelinopathy (i.e., higher variations of L_*u*_) and synaptopathy levels (Figures 2D–F). Moreover, this drop was more significant for SBCs and MSOs. These results show that the disruption of SGN activity due to myelinopathy or synaptopathy has a high downstream impact on the level of phase-locking to sound in the SBC and MSO populations.

**FIGURE 3:**
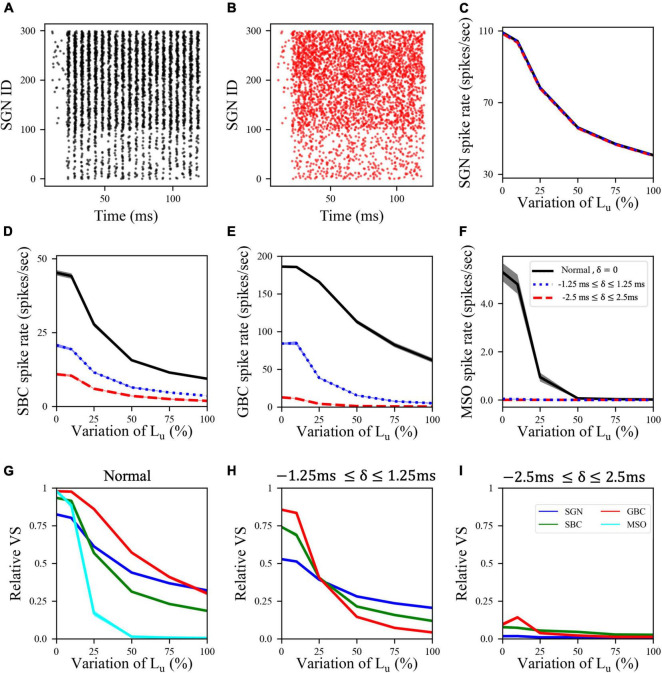
Synchronized synaptic input from SGN cells was necessary to maintain activity rates in downstream cell populations. **(A)** Raster plot showing the activity of a normal SGN population in response to 200 Hz, 50 dB sound stimulus. **(B)** Raster plot where the spike times shown in panel **(A)** are randomized by adding a jitter δ varying between –2.5 and 2.5 ms (high jitter case) to the SGN spike times in panel **(A)**. **(C)** SGN, **(D)** SBC, **(E)** GBC, and **(F)** MSO spike rates for low (–1.25 ms ≤ δ ≤ 1.25 ms) and high jitter and L_u_ variation levels. Here, 0% variation of L_u_ represents a circuit with a homogeneous population of SGNs with 10 μm long L_u_ and 100% variation of L_u_ represents a circuit with a heterogeneous SGN population with 10 μm ≤ L_u_ ≤ 20 μm. **(G–I)** Relative VS of all cell populations at all myelinopathy levels with **(G)** normal (same figure as Figure 2D), **(H)** low jitter and **(I)** high jitter SGN inputs. Increasing randomization of SGN spikes decreased spike rates and relative VS of all cell types at all myelinopathy levels. Note that there is no cyan line in panels **(H,I)** due to no MSO cell firing in low jitter and high jitter cases (dotted-blue and dashed-red lines in panel **(F)**.

In the myelinopathy scenario, the decrease in MSO activity arises from two myelinopathy outcomes at the SGN level: lower and increasingly asynchronous SGN activity. To separately demonstrate the significance of synchronous SGN input on MSO activity, we simulated the activity of all cell types with artificially randomized SGN input. The raster plot of the putative control SGN population in response to 200 Hz, 50 dB sound stimulus is shown in Figure 3A. We then artificially randomized SGN spike times for all myelinopathy levels in order to disrupt synchrony. We jittered each SGN spike time by an amount δ and analyzed two levels of randomization: −1.25 ms ≤ δ ≤ 1.25 ms (i.e., low jitter) and −2.5 ms ≤ δ ≤ 2.5 ms (i.e., high jitter, see Figure 3B for the raster plot of a randomized putative control). Even though SGN spike rates were unchanged (Figure 3C), the activities of downstream cells [SBCs (Figure 3D), GBCs (Figure 3E) and MSOs (Figure 3F)] decreased with increasing jitter, i.e., higher values of δ, for all L_*u*_ variations. Moreover, MSO cell activity was most affected, as SGN inputs with low jitter (dotted-blue line in Figure 3F) and high jitter (dashed-red line in Figure 3F) resulted in no spiking activity in the MSO population for any L_*u*_ variation. Relative vector strengths in all populations decreased with low jitter compared to the control case for all L_*u*_ variations (Figure 3H) and phase-locking to sound was essentially completely abolished in all populations with high jitter regardless of L_*u*_ variation (Figure 3I). The profound effect on MSO spiking provides evidence that not only the level of SGN input but also the degree of its synchronization determines MSO activity.

### Effects of Peripheral Neuropathies on Interaural Time Difference Coding in the Medial Superior Olive

**FIGURE 4:**
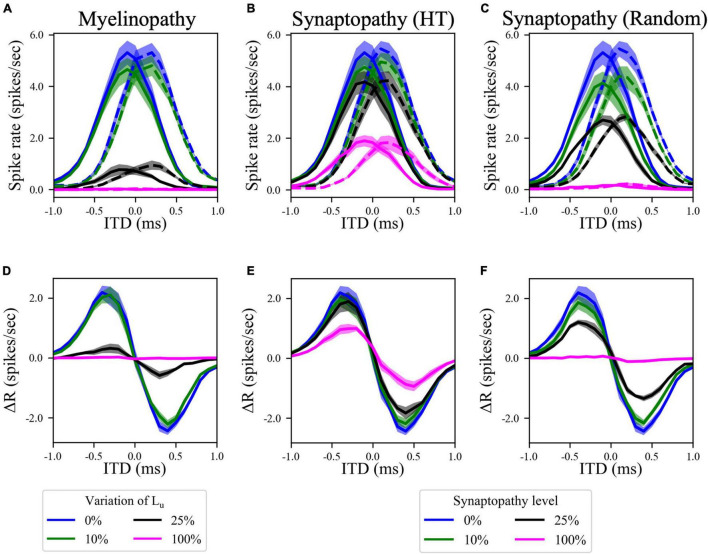
Disruption in the peripheral auditory system decreased MSO spike rates for all ITDs, resulting in smaller differences between left and right MSO activity and hence lower ITD sensitivity. **(A–C)** Spike rates of left (solid lines) and right (dashed lines) MSO cells as a function of ITD for various levels of **(A)** myelinopathy at SGN cells, **(B)** synaptopathy at IHC-HT SGN synapses and **(C)** random synaptopathy in IHC-SGN synapse population. **(D–F)** The difference between left and right MSO firing rates (MSO_left_–MSO_right_) at various levels of **(D)** myelinopathy, **(E)** synaptopathy at HT synapses and **(F)** random synaptopathy. In myelinopathy, 0% variation of L_u_ represents a circuit with a homogeneous population of SGNs with 10 μm long L_u_, and 100% variation of L_u_ represents a circuit with a heterogeneous SGN population with 10 μm ≤ L_u_ ≤ 20 μm. In both synaptopathy scenarios (HT and random), 0% synaptopathy indicates all IHC-SGN synapses are intact. In HT-Synaptopathy a level of 100% means that all IHC-HT SGN synapses are deficient, whereas the same number of synapses are randomly removed in random synaptopathy (1/3rd of all synapses).

Since the horizontal locations of sound sources are encoded by the difference in firing rates between ipsi- and contralateral MSO populations (Grothe and Park, 1998), we simulated MSO activity in both sides for different ITD values, representing the horizontal angle of the sound. Here, ITD > 0 means that sound arrives first to the left ear and ITD < 0 means that sound arrives first to the right ear. For our putative control case, model results showed that MSO activity on one side of the brain reaches a maximum for sounds coming from slightly contralateral positions (Figures 4A–C, blue curves), as the delayed arrival of sound to the ipsilateral ear compensates for the internal delay of the contralateral signal due to the path it travels between the sides of the brain. This asymmetric bell-shaped curve of MSO activity relative to ITDs is critical, as the difference between left and right MSO spike rates encodes the horizontal angle of the sound (Figures 4D–F, blue curves). Simulated increasing degrees of myelinopathy (Figure 4A) and both synaptopathy scenarios (Figures 4B,C) caused a decrease in the peak of this bell-shaped curve, leading to lower rate differences between left and right MSO cells (Figures 4D–F), that, in turn, result in lower ITD sensitivities. This decrease was more pronounced in random synaptopathy than in HT synaptopathy, which is in agreement with Figure 2, showing that all cell types were more affected by random synaptopathy (Figure 2C) than HT synaptopathy (Figure 2B).

### Resonance of Medial Superior Olive Cells Compensates for Effects of Peripheral Neuropathies

**FIGURE 5:**
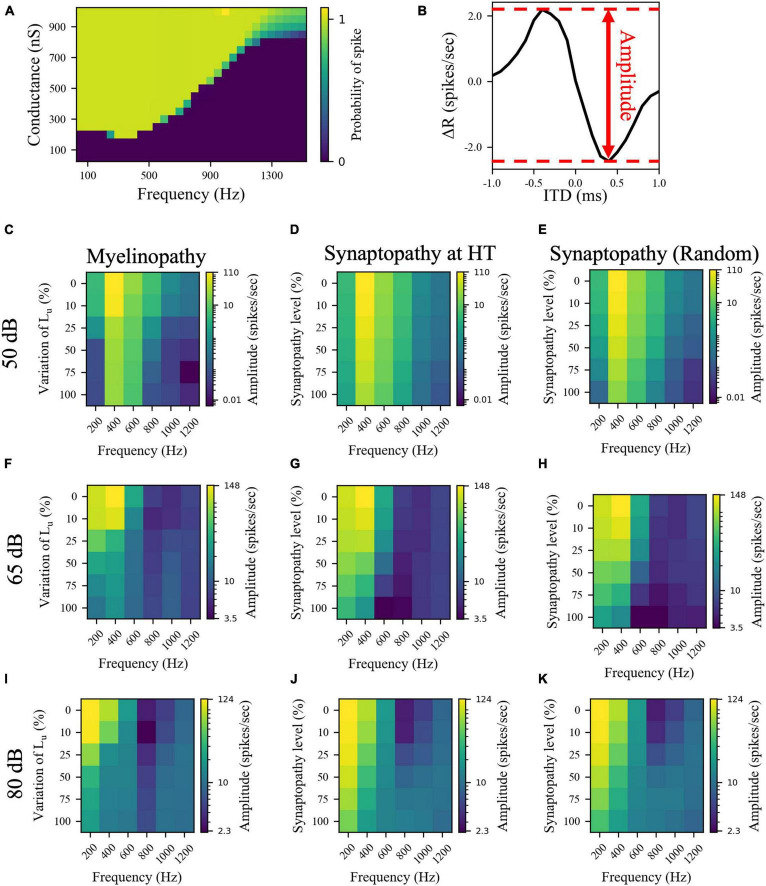
The effects of different peripheral auditory disruption conditions on the activity of MSO cells were less pronounced at the sound frequencies where MSO cells show resonant responses. **(A)** Spike probability (color bar) of individual MSO cells with varying synaptic conductances (Y-axis) in response to different sound frequencies (X-axis). Results provide evidence of resonant behavior for sound frequencies of ∼ 300 Hz. **(B)** The amplitudes in panels **(C–K)** are the distance between the peak and the trough of ΔR, which is the difference between the activities of left and right MSO cells for different ITDs. **(C–K)** The amplitudes of ΔR in response to 50 dB **(C–E)**, 65 dB **(F–H)**, and 80 dB **(I–K)** sound stimuli at frequencies varying from 200 to 1,200 Hz in peripheral auditory disruption scenarios of various levels. In myelinopathy **(C,F,I)**, 0% variation of L_u_ represents a circuit with a homogeneous population of SGNs with 10 μm long L_u_ and 100% variation of L_u_ represents a circuit with a heterogeneous SGN population with 10 μm ≤ L_u_ ≤ 20 μm. In both synaptopathy scenarios [HT **(D,G,J)** and random **(E,H,K)**], 0% synaptopathy indicates all IHC-SGN synapses are intact. Synaptopathy level of 100% means all IHC-HT SGN synapses are deficient in HT synaptopathy, whereas the same number of synapses are randomly removed in random synaptopathy (1/3rd of all synapses).

The asymmetric bell-shaped curves in Figures 4A–C are hallmarks of MSO cell activity resulting from their ability to detect coincident subthreshold presynaptic signals with a high temporal precision (Harper and McAlpine, 2004). Previously, this has been shown to stem from their phasic firing properties, meaning that a step stimulus current evokes only a single spike in MSO cells (Svirskis et al., 2002; Gai et al., 2010; Meng et al., 2012). Neurons with phasic firing patterns have been proven to have frequency-dependent firing thresholds, leading to resonant behaviors (Meng et al., 2012). To assess the significance of resonant responses on different peripheral auditory deficit scenarios, we also explored the resonance properties of the implemented MSO neuron model. To do this, we stimulated a single MSO cell with modified synaptic currents (see “Materials and Methods”), varying their frequency and synaptic strengths (conductances) to calculate the probability that an incoming stimulus generates a spike. The MSO cell exhibited resonance properties with a resonant frequency around 300 Hz, since the threshold conductance needed for spike generation was the lowest at that frequency and it increased with higher and lower frequencies (Figure 5A). This V-shaped colormap (Figure 5A) agrees with experimental (Remme et al., 2014; Mikiel-Hunter et al., 2016) and modeling (Meng et al., 2012; Remme et al., 2014; Mikiel-Hunter et al., 2016) studies that report that MSO cells have a resonant frequency of ∼300 Hz.

**FIGURE 6:**
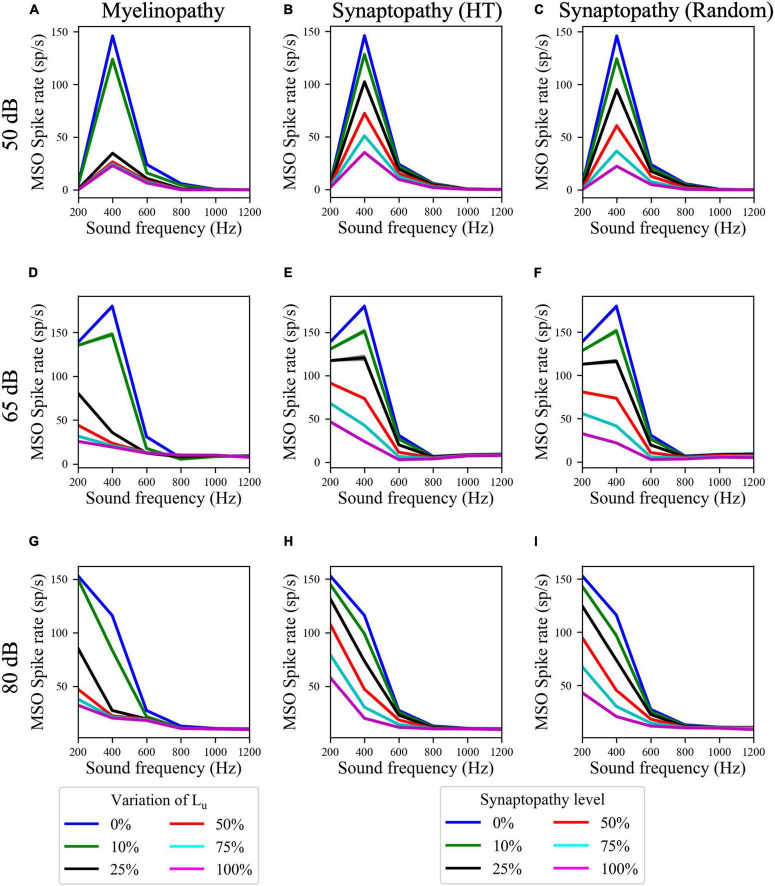
Resonance properties of MSO cells resulted in higher MSO spike rates at resonant frequencies for all peripheral auditory disruption conditions. **(A–I)** MSO spike rates in response to 50 dB **(A–C)**, 65 dB **(D–F)**, and 80 dB **(G–I)** sound stimuli of frequencies varying from 200 to 1,200 Hz in case of myelinopathy **(A,D,G)**, synaptopathy at HT **(B,E,H)** and random synaptopathy **(C,F,I)**. Note that the resonant frequency (frequency with the highest MSO activity) decreased with increasing sound levels.

Next, to determine whether these deficits have differential outcomes on ITD sensitivities of MSOs at resonant compared to non-resonant sound frequencies, we simulated the SOC circuit model with peripheral auditory deficits in response to varying frequencies of sound stimuli. We quantified MSO cells’ ITD sensitivities by the amplitude of the difference between left and right MSO firing rates, ΔR, as a function of ITDs (Figure 5B) and their activity measured by their spike rates (Figure 6). The amplitudes (Figures 5C–K) and the spike rates (Figure 6) of our putative control (0% L_*u*_ variation or 0% synaptopathy) were much higher in response to sound around the resonant frequency (∼300 Hz). However, the sound frequency that resulted in this peak mean activity/ITD sensitivity decreased with increasing sound level from ∼400 Hz at 50 dB to ∼200 Hz at 80 dB. This outcome may stem from higher neurotransmitter release rates from IHCs in response to higher sound levels. Myelinopathy and both synaptopathy scenarios had similar effects on ITD sensitivities (Figures 5C–K) and MSO activities (Figure 6): both measures decreased for all peripheral auditory deficit scenarios, showing graded decreases with increased neuropathy levels for sound stimuli at all frequencies. However, the decreases in ITD sensitivity and MSO spike rate were not as severe for sound stimuli at the resonant frequencies compared to other frequencies.

### Effects of Globular Bushy Cells-Mediated Inhibition on Interaural Time Difference Coding in the Medial Superior Olive

Our SOC model includes GBC-mediated inhibition to MSO cells, however, competing theories exist as to the role of this inhibition for ITD coding in the MSO (Roberts et al., 2013; Myoga et al., 2014). To understand the effect of binaural inhibitory signals to MSO cells, we simulated a model where the input coming from GBCs was removed by modifying Eq. 31, such that:


$$I_{syn,i}\left(t,V_{m}\right)=0$$

First, we simulated MSO activity of our putative control case in response to various sound stimuli and compared the properties of ΔR functions of MSO cells with and without GBC-mediated inhibition. To quantify differences in responses, we used two measures: best ITD difference and amplitude difference (Supplementary Figure 1A). We defined best ITD difference as the difference in MSO cells’ best ITDs between the models with and without inhibition [(best ITD)_*without inhibition*_ − (best ITD)_*with inhibition*_], where best ITD is the ITD value at which MSO cells exhibit the highest activity, i.e., ΔR has the highest value. Results showed that eliminating inhibition does not shift the best ITD significantly (Supplementary Figure 1B). Furthermore, differences of ΔR amplitudes (see Figure 5B for the definition of amplitude) between the two models [(Amplitude)_*without inhibition*_ − (Amplitude)_*with inhibition*_] demonstrated that GBC-mediated inhibition only slightly modulated MSO activity, with bigger changes in response to sound stimuli closer to the resonant frequencies, ∼300 Hz (Supplementary Figure 1C). Finally, we simulated myelin defects in the no-inhibition model and observed the same effects of myelinopathy as in the model with GBC-mediated inhibition, specifically MSO activity was decreased with higher levels of L_*u*_ variation and this decrease was less-pronounced for sound stimuli with near-resonant frequencies (Supplementary Figure 2).

## Discussion

In this study, we built a computational model of mammalian brainstem auditory circuits to understand the impact of peripheral neural deficits, such as SGN myelin defects or loss of IHC-SGN synapses, on binaural auditory processing. Specifically, we explored how the activity of SGNs, cochlear nucleus cells (SBCs and GBCs) and MSO cells are affected by varying degrees of demyelination and synaptopathy of auditory nerve and IHC-SGN synapses, respectively. Motivated by experimental results in animal models, we modeled the degree of demyelination by increasing the range of L_*u*_, the length of the initial unmyelinated segment in SGN axons (Wan and Corfas, 2017), and the degree of synaptopathy by removing IHC-SGN synapses (Kujawa and Liberman, 2009; Furman et al., 2013; Sergeyenko et al., 2013). Model results provided evidence that the activity of an SGN population and the level of phase-locking to the sound stimulus drops with larger ranges of L_*u*_ values and higher degrees of synaptopathy, resulting in decreased firing rates and relative vector strength (VS) of cells downstream of SGNs, i.e., SBCs, GBCs, and MSOs (Figure 2). In synaptopathy conditions, these disrupted spiking patterns of cochlear nucleus and MSO cells stemmed purely from loss of SGN firing activity. However, myelinopathy was found to have two effects on SGN activity patterns: it decreased both the rates and the degree of synchrony of SGN spikes (Budak et al., 2021). To explore solely the effect of synchronous activity of SGN fibers, we artificially added jitter to SGN spikes without decreasing the amount of activity. Model results showed that higher levels of SGN jitter affected the activity and relative VS of downstream cells significantly (Figure 3). This confirms the hypothesis that synchronous activity of SGN fibers is essential for proper cochlear nucleus and MSO activity patterns. Specifically, multiple SGN inputs need to arrive at SBCs within a short time window to generate SBC activity, therefore desynchronized SGN activity reduces the probability of SBC firing. Likewise, lower SBC activity decreases the chance of coincident binaural excitatory input to the MSO, leading to lower MSO firing rates.

Since the difference in MSO firing rates between the left and right MSO is critical for the detection of the horizontal angle of sound sources, lower activity levels in both MSOs, as we observed in all peripheral auditory deficit scenarios we studied, decreases this difference (Figure 4), presumably causing binaural processing and sound localization deficits. These results are in line with behavioral human studies, which provided evidence that myelin defects affecting the auditory nerve or auditory brainstem generate problems in locating sound (Rance et al., 2012; Furst and Levine, 2015; Choi et al., 2018). In addition, synapse loss in the cochlea has been associated with degraded temporal coding in the auditory nerve and brainstem (Bharadwaj et al., 2015) and poorer performance in sound localization tasks (Bharadwaj et al., 2015; Bernstein and Trahiotis, 2016; Prendergast et al., 2017; Eddins et al., 2018).

As MSO cells are known to have resonance properties due to their phasic firing patterns (Svirskis et al., 2002; Gai et al., 2010; Meng et al., 2012), we investigated how effects of peripheral neuropathy may differ at resonant compared to non-resonant frequency sound stimuli. While all neuropathy conditions decreased measures of MSO activity and ITD sensitivity (ΔR amplitude), the deficits were less severe at resonant frequencies. Furthermore, results showed that the disruptive effect of all peripheral auditory neuropathy scenarios could be compensated for by the resonance effect, as the activities of MSO cells were still reasonably high in case of higher levels of synaptopathy or myelinopathy conditions in response to sound stimuli at resonant frequencies (Figures 5C–K, 6). In contrast, the MSO activities in response to sound stimuli with frequencies higher or lower than the resonant frequency were already low in the control case, therefore, peripheral auditory deficits led to significant degradation of MSO activity.

In addition, a counterintuitive effect arises in response to higher frequency sounds at higher levels (80 dB SPL, 800 and 1,000 Hz sound stimuli) where the amplitude of ΔR seemed to increase for increasing levels of neuropathies (Figures 5I–K), even though the MSO spike rates did not show the same effect (Figures 6G–I). This counterintuitive outcome may stem from the fact that MSO cells were overly stimulated at higher sound levels, resulting in smaller differences between left and right MSO spike rates as a function of varying ITDs. However, introducing peripheral neuropathies resulted in a decrease in the amount of input MSO cells received, causing a more significant difference between both MSO spike rates for varying ITDs, even though the absolute rate of MSO activity decreased for all ITD levels.

The compensation for peripheral auditory deficits by MSO cells’ resonance properties would only be significant if these deficits occurred at the SGN fibers having CFs corresponding to resonant frequencies of MSOs. There is no study to our knowledge that investigates the CFs of SGN fibers with myelin defects. However, several animal studies provided evidence that hidden hearing loss occurs as a result of synaptopathy at IHC’s with high CF [>10 kHz in mice (Kujawa and Liberman, 2009), >2 kHz in guinea pigs (Furman et al., 2013), and rhesus monkeys (Valero et al., 2017)]. As MSO cells are not responsive to sound frequencies >1.5 kHz, synaptopathy scenarios at IHC-SGN synapses with high CFs may be more effective on circuits that respond more to high frequency sounds. For instance, like MSO cells, lateral superior olive (LSO) cells are located in SOC, but unlike MSO cells, they are responsible for localizing high frequency sounds using interaural level difference (ILD) cues (Park et al., 2004). Therefore, as a future direction, modeling LSO circuits and assessing ILD sensitivity of LSO cells in synaptopathy scenarios may also give us insight into the mechanisms of binaural deficits caused by synaptopathies.

Changes in the phases of firing, relative to the sound wave, of cells upstream of MSO may also contribute to auditory processing deficits. Model results supported this effect as shown by the significant degradation of MSO activity when SGN spike times were jittered (Figure 3). While those results analyzed variations in the phase-locking to the sound wave, as measured by the VS, the possibility remains that the actual phase angle of spikes relative to the sound wave may be disrupted by SGN myelinopathy and synaptopathy. To investigate this, we further computed average phase angles of spikes, relative to sound waves, of all cell types in our SOC circuit model in response to 50 dB SPL sound stimuli with frequencies of 200 Hz (Supplementary Figure 3), 400 Hz (Supplementary Figure 4), and 600 Hz (Supplementary Figure 5) for both myelinopathy (Supplementary Figures 3A–5A) and HT synaptopathy (Supplementary Figures 3B–5B) scenarios. In the SGN, SBC, and GBC populations, we didn’t observe any significant changes in average phase angles for either neuropathy scenario in response to any frequency sound stimuli. Interestingly, the phase of firing of MSO cells increased significantly for higher myelinopathy levels in response to 400 Hz 50 dB sound stimuli, the frequency closest to the resonant frequency (Supplementary Figure 4A). This result may arise from the resonance properties of MSO cells at 400 Hz, where the absolute decrease in spike rates is the highest for higher levels of myelinopathy (Figure 6A), significantly affecting the average firing phases of MSO cells. In myelinopathy, MSO cells only fire in the first few sound cycles (Supplementary Figure 4M) and therefore lose the subsequent spikes that fire at earlier phases relative to sound due to the gradual phase shift over time that is apparent in the putative control (Supplementary Figure 4L). The loss of only low-phase spikes results in an increase in the average phase angle of firing in MSO cells. Further investigation on circuits downstream of MSO cells (e.g., inferior colliculus, medial geniculate body, etc.) would be useful to better understand the effect of myelinopathy-induced phase angle increases on auditory perceptual deficits. This analysis showing minimal effects of peripheral neuropathy on spike phase angle, relative to the sound wave, suggests that the primary mechanism underlying binaural deficits obtained in the model is the loss of excitatory signaling from SGN fibers, that results in lower coincident signals downstream of SGNs, thus decreasing activity levels in cochlear nucleus and MSO cells.

In our SOC circuit, we included binaural inhibitory inputs that MSO cells receive from GBCs via relays through the medial and lateral nuclei of the trapezoid body (MNTB and LNTB). However, the function of these inhibitory signals has remained controversial over the years. In particular, some studies suggest that they play a role in shifting the best ITD of MSO cells toward more contralateral ITD values, contributing to the ITD sensitivity of MSO cells (Myoga et al., 2014), whereas other studies claim that inhibition does not shift the window for detecting binaural coincidence, but only reduces the levels of excitation to refine ITD sensitivity or to preserve ITD sensitivity at higher sound intensities (Roberts et al., 2013). Our simulations of the SOC circuit with GBC cells removed addressed these competing hypotheses (Supplementary Figure 1). In putative control conditions (either 0% L_*u*_ variation or 0% synaptopathy) in response to sound stimuli at various sound levels and frequencies, model results showed that best ITDs did not significantly shift (Supplementary Figure 1B), but that ITD sensitivity, measured by the amplitude ΔR, was modulated (Supplementary Figure 1C), as hypothesized in Roberts et al. (2013).

In conclusion, our model results showed that mechanisms underlying HHL in animal models, such as myelin defects at SGN fibers or synapse loss at IHC-SGN synapses, may have significant effects on downstream auditory cell responses, such as MSO cell activity, which plays a crucial role in sound localization. Results indicate that loss of SGN spiking activity as well as potentially asynchronous spike timing contribute to a degradation of ITD sensitivity and coding in the MSO. Model results predict that the primary mechanism inducing binaural auditory processing deficits in myelinopathy and synaptopathy conditions is a decrease in SGN firing activity.

These results may provide a reasonable explanation for human auditory deficits where audiometric thresholds are normal but encoding and processing of binaural cues are degraded. In addition, this study may give us insight into possible treatments for HHL scenarios to overcome these binaural processing deficits. To better elucidate the mechanisms of perceptual deficits resulting from peripheral auditory neuropathies, effects on LSO activity patterns should also be investigated, as LSO cells are responsible for localizing high frequency sounds (Park et al., 2004). Our model results additionally provided evidence that peripheral synaptopathy and myelinopathy cause decreased neuronal activity throughout brainstem auditory circuitry, namely all the way from SGN fibers to MSO cells. Such chronic decreases in neuronal activity may result in homeostatic plasticity downstream of the circuit, as sensory deprivation is already known to cause homeostatic plasticity to compensate for decreased neuronal activity in the brain (Turrigiano et al., 1998; Turrigiano, 2008; Bishop and Zito, 2013; Whitt et al., 2014), including in the auditory system (Teichert et al., 2017). Therefore, studying the impact of these peripheral deficits on downstream auditory circuits from midbrain to auditory cortex may shed light into homeostatic plasticity mechanisms and its consequences such as the perception of tinnitus (Yang et al., 2011).

## Materials and Methods

### Peripheral Auditory System Model

The activity of the peripheral auditory system is simulated by a guinea pig model described in Budak et al. (2021). Even though gerbils are widely used models for MSO investigations due to their responsiveness to low-frequency sound stimuli that give rise to detectable ITDs (Grothe and Neuweiler, 2000), guinea pigs have comparable SGN responses to low frequency sound as gerbils, making the guinea pig model a good fit to our study. This model simulates the responses of various parts of the ear, starting from the middle ear to IHC-SGN synapses, in response to a sound wave characterized by its frequency and sound pressure level (SPL in dB) (Sumner et al., 2002; Steadman, 2018; Steadman and Sumner, 2018). It outputs the probability of neurotransmitter release from IHCs to IHC-SGN synapses, which is used to determine a Poisson process of release events from each IHC-SGN synapse.

### Spiral Ganglion Neurons Fiber Model

A compartmental model for peripheral axons of SGN fibers is modeled as described in Budak et al. (2021). Briefly, each SGN fiber has an initial unmyelinated segment of length L_*u*_ and a heminode of length L_*h*_. The putative control is identified as an SGN population with all fibers having L_*u*_ of 10 μm and L_*h*_ of 1 μm. Motivated by experimental observations (Wan and Corfas, 2017), we model myelinopathy by forming populations of SGNs with heterogeneous L_*u*_ values where we increased L_*u*_ variation up to a range of 10–20 μm. We denote a homogeneous population with our putative control (*L_*u*_* = 10 μm) as 0% variation of *L*_*u*_, and the heterogeneous population with *L*_*u*_ values distributed uniformly between 10 and 20 μm as 100% variation of *L*_*u*_. We also model synaptopathy by removing IHC-SGN synapses, i.e., the synaptopathy level represents the ratio of the number of removed synapses with the number of the defined synapse population (0% synaptopathy when all synapses are present and 100% when all synapses are removed).

Each IHC-SGN synapse is connected to one SGN fiber. Each release event determined by the peripheral auditory system model triggers a post-synaptic response at the corresponding SGN fiber that is modeled as an external current pulse (Budak et al., 2021). This way, spike trains of each SGN in response to sound stimuli are generated. For simplicity, we only consider the SGNs that have the same characteristic frequency (CF) as the frequency of the sound stimulus.

We include three different types of IHC-SGN synapses based on the response to varying sound stimuli levels: low-threshold (LT), medium-threshold (MT), and high-threshold (HT) synapses. We denote the SGNs based on the type of synapses they are connected to (e.g., an SGN fiber connected to a LT synapse is called a LT SGN). In each side, we model 100 low-threshold (LT), 100 medium-threshold (MT), and 100 high-threshold (HT) SGNs.

### Cochlear Nucleus Network Structure

Spiral ganglion neurons that are activated by sound stimuli relay this signal to the cochlear nucleus. SBCs and GBCs in the cochlear nucleus receive excitatory inputs from multiple ipsilateral SGNs [2–4 to SBCs and 9–69 to GBCs (Sento and Ryugo, 1989; Liberman, 1991; Ryugo and Sento, 1991; Spirou et al., 2005)], leading to higher phase-locking with the sound signal. MSO cells receive binaural excitatory inputs directly from SBCs and binaural inhibitory inputs from GBCs via one of two relay points, the medial nucleus of the trapezoid body (MNTB) and the lateral nucleus of the trapezoid body (LNTB), which relay input from contralateral and ipsilateral GBCs, respectively.

In our reduced cochlear nucleus circuit model, we modeled SBC, GBC, and MSO cell populations, with each population containing 300 neurons (Figure 1). SBC neurons send direct excitatory input to MSO cells and, for simplicity, we assumed GBCs send inhibitory signals directly to MSO cells, as in Encke and Hemmert (2018). Each SBC receives four and each GBC receives 40 excitatory inputs from the population of ipsilateral SGNs. MSO is the first region of binaural integration in the auditory circuit, and each cell in the MSO receives six excitatory inputs from SBCs in each side and three inhibitory inputs from GBCs in each side (Figure 1). Because in the biological circuit MNTB neurons convert input from GBCs into spikes with extremely high reliability and temporal precision (Mc Laughlin et al., 2008; Lorteije et al., 2009), we decided that explicitly incorporating MNTB neurons into the model would have no meaningful effect on the results since they essentially act as biological relays. Our decision here is in line with previous models of the MSO (Encke and Hemmert, 2018). Moreover, very little is known about the *in vivo* function of LNTB neurons (Guinan et al., 1972a,b; Tsuchitani, 1977). Because of this, it is not clear how LNTB neurons should be modeled. Therefore, for the sake of simplicity and symmetry in how the ipsilateral and contralateral inhibitory inputs were treated, we decided to model inhibitory input to the MSO as coming directly from GBCs.

### Node Dynamics of Spherical Bushy Cells, Globular Bushy Cells, and Medial Superior Olive

For neuron dynamics, we implemented previously developed models used in a cochlear nucleus circuit model of gerbils (Encke and Hemmert, 2018). Here, GBC and MSO cells are modeled as single compartment Hodgkin-Huxley type models, with parameters and ion channels adjusted to experimental observations in gerbil models (Encke and Hemmert, 2018). Since both SBC and GBC cells have comparable dynamics, only varying in morphology and the number of inputs from SGN fibers (Kuenzel, 2019), SBC cells are modeled with the same node dynamics as GBC cells but different numbers and strengths of excitatory synaptic inputs from SGNs.

The membrane potentials of SBCs and GBCs are modeled as:


(1) $$\begin{array}{c} C_{m}\frac{dV_{m}}{dt}=-(g_{l}\left(V_{m}-E_{rest}\right)+g_{Na}m^{3}h\left(V_{m}-E_{Na}\right)\\ \;+g_{KHT}\left(0.85n^{2}+0.15p\right)\left(V_{m}-E_{K}\right)+g_{KLT}w^{4}z\left(V_{m}-E_{K}\right) \end{array}$$

where *C*_*m*_ is the membrane capacitance, *g*_*l*_ is the leak conductance, *g*_*Na*_ is the Na^+^ conductance, *g*_*KHT*_ and *g*_*KLT*_ are high and low threshold K^+^ conductances, respectively, and *g*_*h*_ is the conductance of the hyperpolarization-activated cation current, or *H* current. *E*_*rest*_ stands for the resting membrane potential and *E*_*x*_ represents the Nernst potentials of each ionic current *x* (for *x* = Na^+^, K^+^, and H) (Table 1). The variables *m, h, n*, *p, w, z*, and *r* are the voltage dependent conductance gating variables expressed as:


(2) $$\frac{di}{dt}=\alpha_{i}\left(V_{m}\right)\left(1-i\right)-\beta_{i}\left(V_{m}\right)i\;fori=m,h$$

and


(3) $$\frac{dj}{dt}=\frac{j_{\infty }-j}{j_{\tau }}forj=n,p,w,z,r$$

where,


(4) $$\alpha_{m}\left(V_{m}\right)=\frac{1.872\left(V_{m}+49\right)}{1-e^{\frac{-\left(V_{m}+49\right)}{3}}}$$

(5) $$\beta_{m}\left(V_{m}\right)=\frac{-2.08\left(V_{m}+58\right)}{1-e^{\frac{\left(V_{m}+58\right)}{20}}}$$

(6) $$\alpha_{h}\left(V_{m}\right)=\frac{12.48}{1+e^{\frac{\left(V_{m}+68\right)}{3}}}+\frac{25.344}{1+e^{V_{m}+61.3}}$$

(7) $$\beta_{h}\left(V_{m}\right)=\frac{18.72}{1+e^{\frac{-\left(V_{m}+21\right)}{10}}}$$

(8) $$w_{\infty }\left(V_{m}\right)={\left(1+e^{\frac{-\left(V_{m}+48\right)}{6}}\right)}^{-0.25}$$

(9) $$\tau_{w}\left(V_{m}\right)=1.5+\frac{100}{6e^{\frac{\left(V_{m}+60\right)}{6}}+16e^{\frac{-\left(V_{m}+60\right)}{45}}}$$

(10) $$z_{\infty }\left(V_{m}\right)=0.5+\frac{1}{2\left(1+e^{\frac{\left(V_{m}+71\right)}{10}}\right)}$$

(11) $$\tau_{z}\left(V_{m}\right)=50+\frac{1000}{e^{\frac{\left(V_{m}+60\right)}{20}}+e^{\frac{-\left(V_{m}+60\right)}{8}}}$$

(12) $$r_{\infty }\left(V_{m}\right)={\left(1+e^{\frac{\left(V_{m}+76\right)}{7}}\right)}^{-1}$$

(13) $$\tau_{r}\left(V_{m}\right)=25+\frac{100000}{237e^{\frac{\left(V_{m}+60\right)}{12}}+17e^{\frac{-\left(V_{m}+60\right)}{14}}}$$

(14) $$n_{\infty }\left(V_{m}\right)={\left(1+e^{\frac{-\left(V_{m}+15\right)}{5}}\right)}^{-0.5}$$

(15) $$\tau_{n}\left(V_{m}\right)=0.7+\frac{100}{11e^{\frac{\left(V_{m}+60\right)}{24}}+21e^{\frac{-\left(V_{m}+60\right)}{23}}}$$

(16) $$p_{\infty }\left(V_{m}\right)={\left(1+e^{\frac{-\left(V_{m}+23\right)}{6}}\right)}^{-1}$$

(17) $$\tau_{p}\left(V_{m}\right)=5+\frac{100}{4e^{\frac{\left(V_{m}+60\right)}{32}}+5e^{\frac{-\left(V_{m}+60\right)}{22}}}.$$

**TABLE 1:** Parameters for spherical bushy cells (SBC), globular bushy cells (GBC), and medial superior olive (MSO) cells.

Parameters		SBC/GBC	MSO
C_*m*_ (pF)	Membrane capacitance	12	70
E_*rest*_ (mV)	Resting membrane potential	−65	−55.8
g_*l*_ (nS)	Leak conductance	37	13
E_*Na*_ (mV)	Nernst potential of Na^+^	50	56.2
g_*Na*_ (nS)	Na^+^ conductance	4592.8	3900
E_*K*_ (mV)	Nernst potential of K^+^	−77	−90
g_*KHT*_ (nS)	High threshold K^+^ conductance	35.1	N/A
g_*KLT*_ (nS)	Low threshold K^+^ conductance	367.4	650
E_*h*_ (mV)	Nernst potential of H current	−43	−35
g_*h*_ (nS)	H current conductance	36.7	520
A (nS)	Synaptic strength	13/4.76	*I*_*syn,e*_: 54.37/*I*_*syn,i*_: 5.5

I_*syn*_ is the excitatory synaptic current generated by SGN activity expressed as


(18) $$I_{syn}\left(t,V_{m}\right)=\sum_{i=1}^{N}Ae^{\frac{-\left(t-t_{s_{i}}\right)}{0.2}}\left(V_{m}-E_{ex}\right)$$

where *N* is the number of presynaptic spikes, *t*_*s_i_*_ is the time of the presynaptic SGN spike *i* and *E*_*ex*_ = 0 mV is the reversal potential for excitatory current. *A* is the synaptic strength and equals 4.76 nS for GBCs, as in Encke and Hemmert (2018), and 13 nS for SBC that was adjusted to obtain experimentally observed responses for the activity of gerbil SBC (Kuenzel et al., 2011).

The current balance equation for MSO cells is expressed as:


(19) $$\begin{array}{c} C_{m}\frac{dV_{m}}{dt}=-(g_{l}\left(V_{m}-E_{rest}\right)+g_{Na}m^{3}h\left(V_{m}-E_{Na}\right)+g_{KLT}w^{4}z\\ \left(V_{m}-E_{K}\right)+g_{h}r\left(V_{m}-E_{h}\right)+I_{syn,e}+I_{syn,i}), \end{array}$$

with parameters as in Equation 1 (Table 1). The variables *m, h, w, z*, and *r* are the voltage dependent conductance gating variables that are governed by Equation 3 with steady state activation and time constant functions given by:


(20) $$m_{\infty }\left(V_{m}\right)={\left(1+e^{\frac{-\left(V_{m}+38\right)}{7}}\right)}^{-1}$$

(21) $$\tau_{m}\left(V_{m}\right)=\frac{0.48}{5e^{\frac{\left(V_{m}+60\right)}{18}}+36e^{\frac{-\left(V_{m}+60\right)}{25}}}$$

(22) $$h_{\infty }\left(V_{m}\right)={\left(1+e^{\frac{\left(V_{m}+65\right)}{6}}\right)}^{-1}$$

(23) $$\tau_{h}\left(V_{m}\right)=\frac{19.23}{7e^{\frac{\left(V_{m}+60\right)}{11}}+10e^{\frac{-\left(V_{m}+60\right)}{25}}+0.12}$$

(24) $$w_{\infty }\left(V_{m}\right)={\left(1+e^{\frac{-\left(V_{m}+57.3\right)}{11.7}}\right)}^{-1}$$

(25) $$\tau_{w}\left(V_{m}\right)=\frac{46}{6e^{\frac{\left(V_{m}+75\right)}{12.15}}+24e^{\frac{-\left(V_{m}+75\right)}{25}}+0.55}$$

(26) $$z_{\infty }\left(V_{m}\right)=0.4+0.6{\left(1+e^{\frac{\left(V_{m}+57\right)}{5.44}}\right)}^{-1}$$

(27) $$\tau_{z}\left(V_{m}\right)=12+\frac{240}{e^{\frac{\left(V_{m}+60\right)}{20}}+e^{\frac{-\left(V_{m}+60\right)}{8}}}$$

(28) $$r_{\infty }\left(V_{m}\right)={\left(1+e^{\frac{\left(V_{m}+80.4\right)}{10}}\right)}^{-1}$$

(29) $$\tau_{r}\left(V_{m}\right)=79+e^{\frac{-{\left(V_{m}+61.5\right)}^{2}}{800}}$$

I_*syn,e*_ and I_*syn,i*_ are excitatory and inhibitory synaptic currents received from the SBC and GBC cells, respectively. I_*syn,e*_ is described as


(30) $$I_{syn,e}\left(t,V_{m}\right)=\sum_{i=1}^{N}A\frac{t-t_{s_{i}}-t_{delay}}{0.17}e^{-\left(\frac{t-t_{s_{i}}-t_{delay}}{0.17}\right)}\left(V_{m}-E_{ex}\right),$$

where *N* is the number of presynaptic SBC spikes, *t*_*s_i_*_ is the time of the i-th presynaptic SBC spike, E_*ex*_ = 0 mV is the reversal potential for excitatory current and *A* = 54.37 nS is the synaptic strength. The *t*_*delay*_ is the time required for the signal from SBCs to reach MSO cells, which is 1.5 ms for ipsilateral input and 1.6 ms for contralateral input (Encke and Hemmert, 2018).

I_*syn,i*_ is expressed as:


(31) $$\begin{array}{c} I_{syn,i}\left(t,V_{m}\right)=\\ -\sum_{i=1}^{N}\{\left(A\left(e^{-\left(\frac{t-t_{s_{i}}-t_{delay}}{0.14}\right)}-e^{-\left(\frac{t-t_{s_{i}}-t_{delay}}{1.6}\right)}\right)\right\}\left(V_{m}-E_{in}\right), \end{array}$$

where *N* is the number of presynaptic GBC spikes, *t*_*s_i_*_ is the time of the i-th presynaptic GBC spike, *E*_*ex*_ = −70 mV is the reversal potential for excitatory current and *A* = 5.5 nS is the synaptic strength. The *t*_*delay*_ is the time required for the signal from inhibitory GBCs to reach MSO cells, which is 1.5 ms for ipsilateral input and 1.0 ms for contralateral input (Encke and Hemmert, 2018).

### Relative Vector Strength Measurement

Vector strength (VS) is a measure to determine the degree of phase-locking of spiking in a neuron population to a sound wave. To calculate VS for each neural population, the phase angle θ*_*i*_* relative to the sound wave of each spike *i* fired by cells in the population is first measured as:


(32) $$\theta_{i}=\frac{\sum_{i=1}^{N}\frac{t_{i}-t_{1}}{t_{2}-t_{1}}}{n},$$

where *n* is the number of spikes of a cell population, *t*_*i*_ is the time of each spike *i*, and *t*_1_ and *t*_2_ are the peaks of the sound wave just before and just after *t*_*i*_, respectively. Vector strength (Goldberg and Brown, 1969) is then calculated as:


(33) $$VS=\frac{\sqrt{{\left[\sum_{i=1}^{n}\cos\theta_{i}\right]}^{2}+{\left[\sum_{i=1}^{n}\sin\theta_{i}\right]}^{2}}}{n}.$$

This measure varies between 0 and 1, where 1 means all spikes are phase-locked to the sound wave at the same angle and 0 means no phase-locking to sound.

**FIGURE 7:**
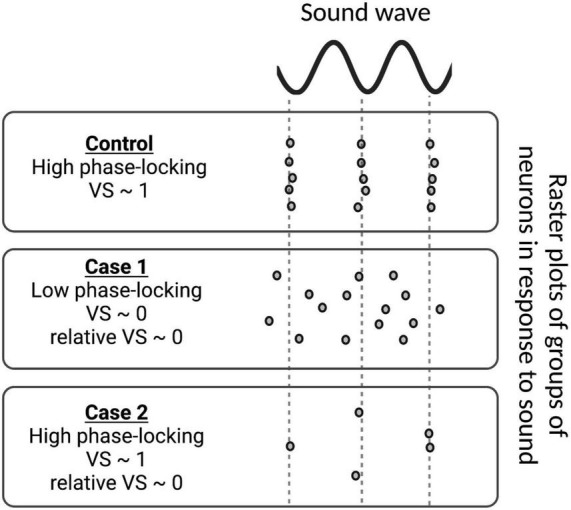
Examples of vector strength (VS) and relative VS measurements for various groups of neurons in response to sound stimulus. In the control case, the spike times of all neurons are phase-locked to the sound wave, i.e., they always fire at the same phase of the sound wave and VS approaches 1. In Case 1, phase-locking is low, resulting in a VS ∼ 0. As the number of spikes is the same as the control in Case 1, VS and relative VS are the same. However, the group of neurons in Case 2 does not fire nearly as much as the control case, leading to low relative VS, even though its VS is high.

A measure of relative VS is derived from VS in order to assess the degree of phase-locking relative to the putative control. It is defined as:


(34) $$RelativeVS=\frac{\sqrt{{\left[\sum_{i=1}^{n}\cos\theta_{i}\right]}^{2}+{\left[\sum_{i=1}^{n}\sin\theta_{i}\right]}^{2}}}{N},$$

where *N* is the total number of spikes in the putative control. As in VS, relative VS also varies between 0 and 1. Here, a relative VS = 1 means perfect phase-locking to sound with the same number of spikes relative to the putative control, whereas a relative VS = 0 happens in case of low phase-locking and/or low number of spikes relative to the control (Figure 7).

### Identifying Resonance Properties

To detect resonance properties in the MSO cell model, we simulated MSO cell responses to direct excitatory synaptic input arriving at various frequencies. We used the same node dynamics of MSO cells described before (see Equations 19–31 and Table 1) and only modified excitatory and inhibitory synaptic input (*I*_*syn,e*_ and *I*_*syn*,_*_*i*_*, respectively, see Equations 30, 31), such that:


$$I_{syn,i}\left(t,V_{m}\right)=0$$

and


$$I_{syn,e}\left(t,V_{m}\right)=A\frac{t-t_{s}\left(t,f\right)}{0.17}e^{-\left(\frac{t-t_{s}\left(t,f\right)}{0.17}\right)}\left(V_{m}-E_{ex}\right)$$

where


$$t_{s}\left(t,f\right)=t-mod\left(t,\frac{1}{f}\right).$$

Here, *t*_*s*_ is the presynaptic spike time, *E*_*ex*_ = 0 mV is the reversal potential for excitatory current, *f* is the frequency of presynaptic spikes, *V*_*m*_ is the membrane potential and *A* is the synaptic strength (synaptic conductance). For the resonance study, we varied *f* and *A*, and calculated spike probabilities (Figure 5) as follows:


(35) $${\text{p}}_{\text{spike}}=\frac{\mathrm{Number}\mathrm{of}\mathrm{MSO}\mathrm{spikes}}{\mathrm{Number}\mathrm{of}\mathrm{synaptic}\mathrm{inputs}}.$$

### Simulations

We simulated the sound-evoked activity of all cell types in response to a sound stimulus pulse of 200 Hz and 50 dB for 100 ms, unless stated otherwise. Our results show the responses averaged over five sound stimuli.

## Conclusion

Some people have difficulty understanding speech in crowded social settings, also known as the “cocktail party problem,” despite having normal hearing thresholds for all sound frequencies. One potential cause of this condition is “hidden hearing loss” (HHL), an auditory disorder resulting from noise exposure, aging or peripheral neuropathy. In this study, we hypothesized that the perceptual deficits caused by HHL arise from sound localization problems due to disrupted inputs to the medial superior olive (MSO), a nucleus in the brainstem that integrates binaural signals to determine the relative timing of sound arrival to both ears and to detect the horizontal angle of the sound source. To explore the impacts HHL has on MSO activity, we simulated MSO circuits that receive signals from both ears affected by two peripheral neuropathies which have been previously shown to cause HHL in animal models: (1) loss of synapses between inner hair cells and auditory nerve fibers, and (2) disruption of auditory-nerve myelin. We provide evidence that both scenarios disrupt the activity of MSO cells correlated with sound localization that may, in turn, result in speech intelligibility deficits. This model may be used to elucidate downstream effects of peripheral neuropathies and to propose possible clinical treatments for HHL.

## Data Availability Statement

The raw data supporting the conclusions of this article will be made available by the authors, without undue reservation.

## Author Contributions

MB performed the research and analyzed the data. MB, MR, KG, GC, VB, and MZ designed the research, wrote the manuscript, contributed to the article, and approved the submitted version.

## Conflict of Interest

The authors declare that the research was conducted in the absence of any commercial or financial relationships that could be construed as a potential conflict of interest.

## Publisher’s Note

All claims expressed in this article are solely those of the authors and do not necessarily represent those of their affiliated organizations, or those of the publisher, the editors and the reviewers. Any product that may be evaluated in this article, or claim that may be made by its manufacturer, is not guaranteed or endorsed by the publisher.
