# Supplementary material for: Binaural Processing Deficits Due to Synaptopathy and Myelin Defects
Source: Front Neural Circuits. 2022 Apr 14;16:856926. doi: 10.3389/fncir.2022.856926 (PMC9050145; doi:10.3389/fncir.2022.856926)
Supplement: Supplementary file 1 [file Data_Sheet_1.pdf]

Supplementary Figures

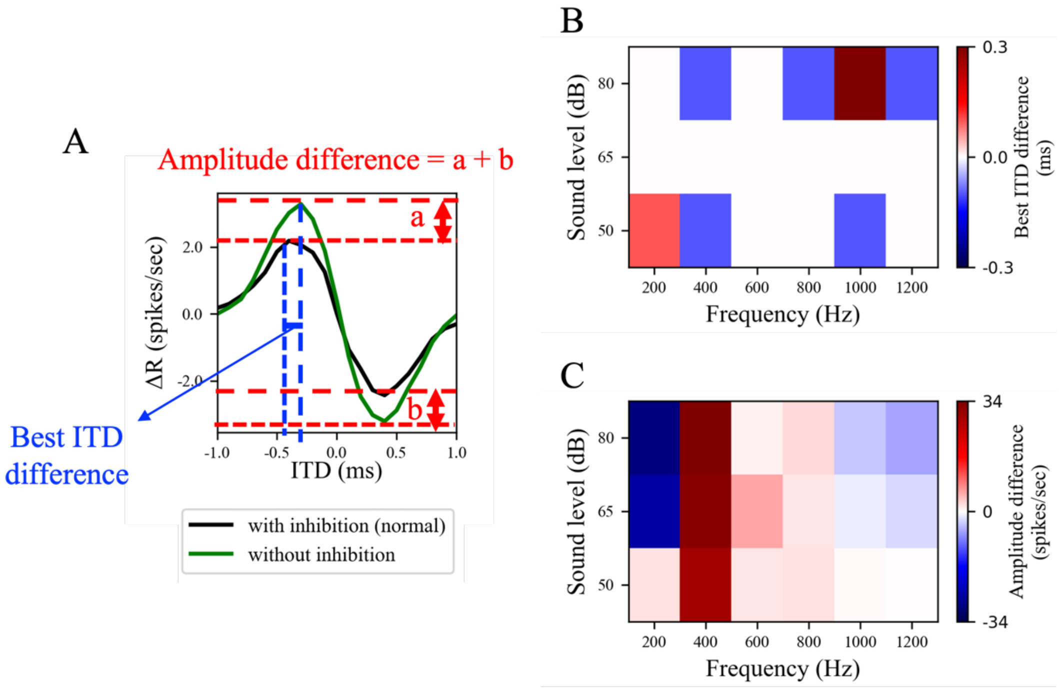

Supplementary Figure 1. Inhibition from GBCs modulates the activity levels of MSO cells without shifting the best ITDs. (A) The best ITD difference measured in (B) is defined by (best ITD)$_{\text{without inhibition}}$ - (best ITD)$_{\text{with inhibition}}$, where best ITD is the ITD value at which MSO cells exhibit the highest activity, i.e., ΔR (the difference between the activities of left and right MSO cells) has the highest value. The amplitude difference measured in (C) is the difference of amplitudes of ΔR (the distance between the peak and the trough of ΔR): (Amplitude)$_{\text{without inhibition}}$ - (Amplitude)$_{\text{with inhibition}}$.(B) Best ITD differences and (C) amplitude differences in response to varying sound levels (50 dB, 65 dB and 80 dB) and sound frequencies (200 Hz to 1200 Hz) show that inhibition does not shift the peak of ΔR, only modulates it.

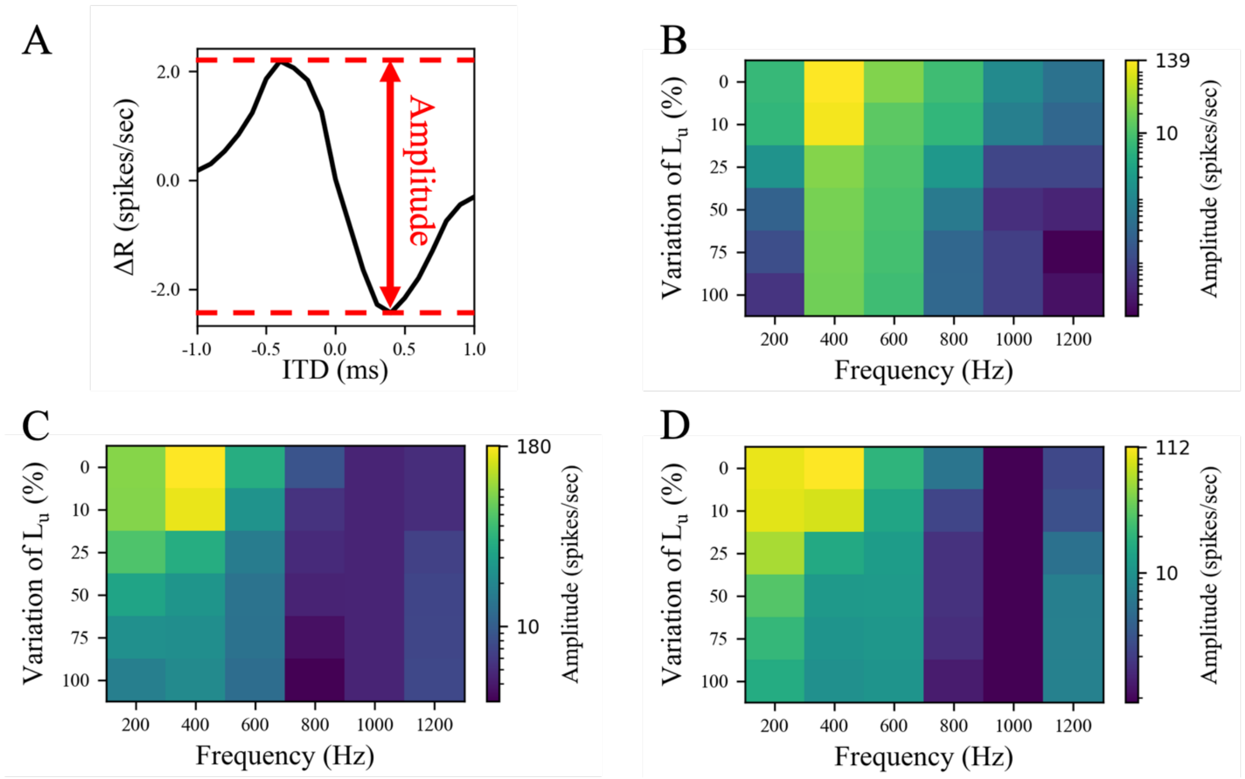

Supplementary Figure 2. MSO circuit model without inhibition (without GBCs) shows similar responses to L_u variation of SGNs such that higher levels of L_u variation gradually decrease MSO activities, with a less-pronounced effect in response to sound stimuli with resonant frequencies (~300 Hz). (A) The amplitudes in (B-D) are the distance between the peak and the trough of ΔR, which is the difference between the activities of left and right MSO cells. (B-D) The amplitudes of ΔR in response to (B) 50dB, (C) 65dB and (D) 80dB sound stimuli of frequencies varying from 200Hz to 1200Hz in case of various levels of L_u variation. 0% variation of L_u represents a circuit with a homogeneous SGN population with 10 μm long L_u and 100% variation of L_u represents a circuit with a heterogeneous SGN population with 10 μm ≤ Lu ≤ 20 μm.

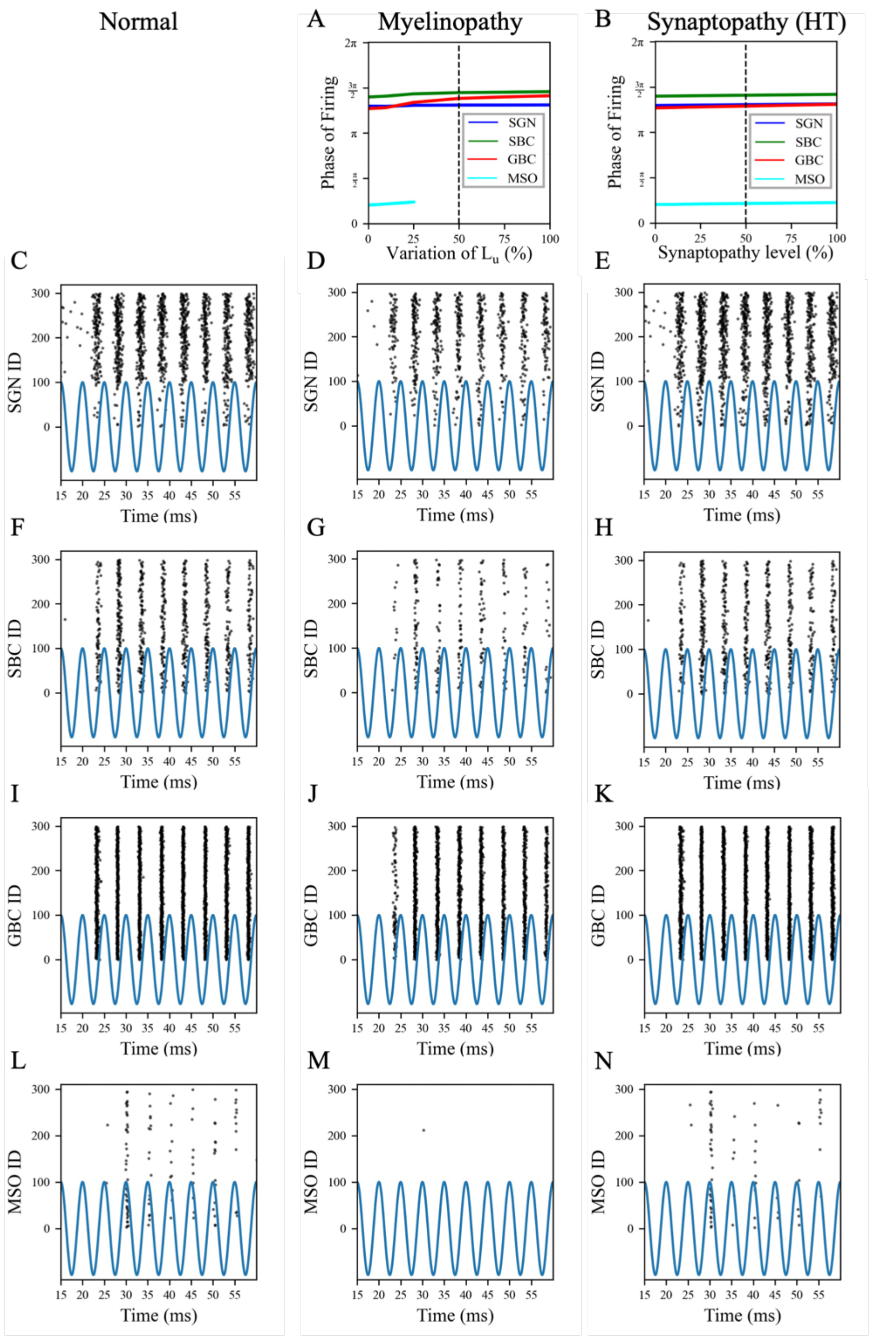

Supplementary Figure 3. Average phases of spikes of all cell types in MSO circuits do not change with higher myelinopathy or synaptopathy levels in response to 200 Hz sound stimulus. Average phases of firing, relative to the sound wave, of cell populations (SGNs, SBCs, GBCs, MSOs) with respect to 200 Hz sound stimulus in cases of various (A) myelinopathy and (B) HT synaptopathy levels, where dashed lines in both panels indicate the level of myelinopathy/synaptopathy of the raster plots below (D, G, J, M for myelinopathy and E, H, K, N for synaptopathy). The raster plots of 300 SGNs (C, D and E), 300 SBCs (F, G, H), 300 GBCs (I, J, K) and 300 MSOs (L, M and N) of the control (C, F, I and L), i.e., 0% L_u variation or 0% synaptopathy, 50% L_u variation (D, G, J and M) and 50% synaptopathy (E, H, K and N), with 200 Hz sound waves superimposed on the raster plots for phase comparison. Note that MSOs do not fire for L_u variation higher than 25%, therefore, cyan curve is not defined for variation of L_u > 25%.

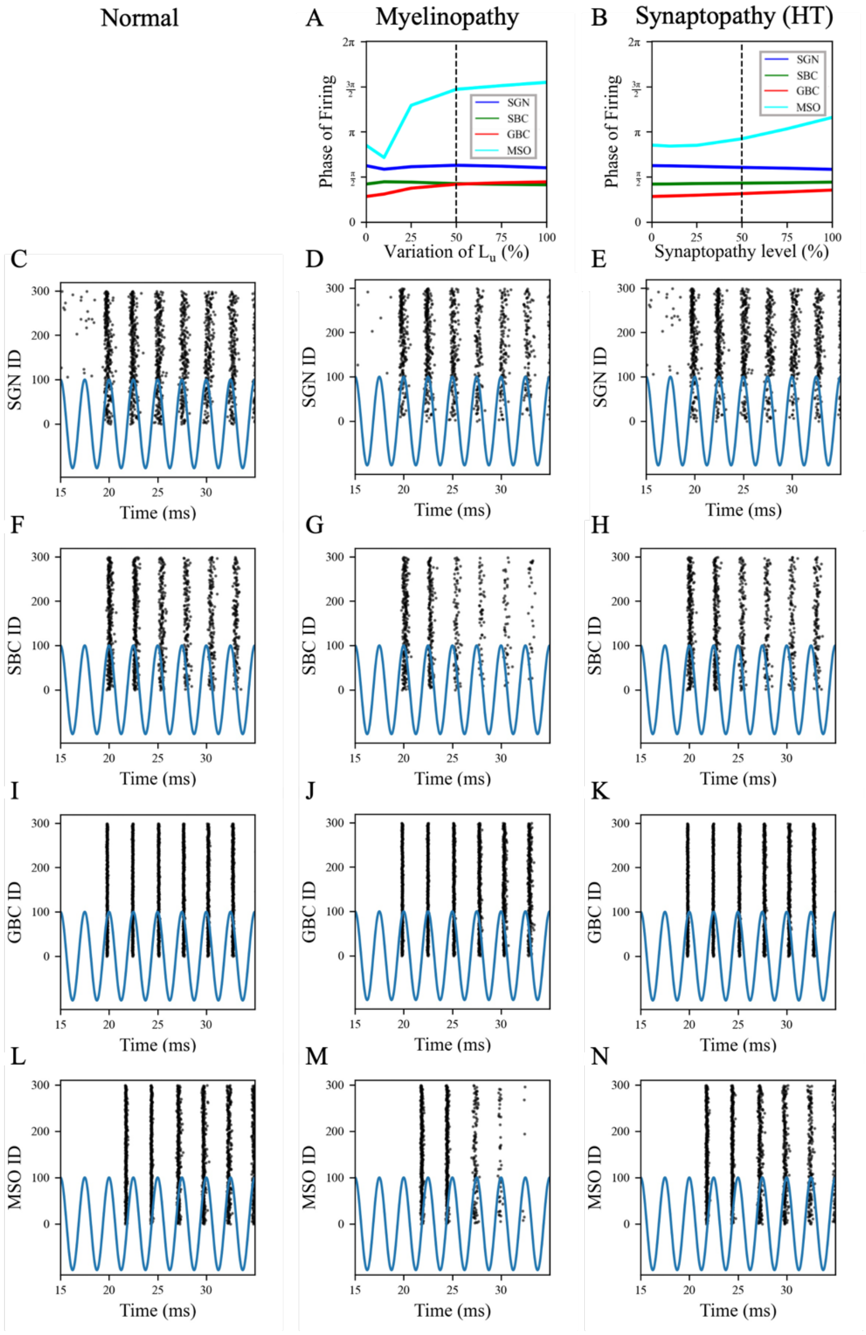

Supplementary Figure 4. Average phases of spikes of SGN, SBC and GBC cells in MSO circuits do not change with higher myelinopathy or synaptopathy levels in response to 400 Hz sound stimulus, while average phase of MSO cells increases for higher L_u variation. Average phases of firing, relative to the sound wave, of cell populations (SGNs, SBCs, GBCs, MSOs) with respect to 400 Hz sound stimulus in case of various (A) myelinopathy and (B) HT synaptopathy levels, where dashed lines in both panels indicate the level of myelinopathy/synaptopathy of the raster plots below (D, G, J, M for myelinopathy and E, H, K, N for synaptopathy). The raster plots of 300 SGNs (C, D and E), 300 SBCs (F, G, H), 300 GBCs (I, J, K) and 300 MSOs (L, M and N) of the control (C, F, I and L), i.e., 0% L_u variation or 0% synaptopathy, 50% L_u variation (D, G, J and M) and 50% synaptopathy (E, H, K and N), with 200 Hz sound waves superimposed on the raster plots for phase comparison.

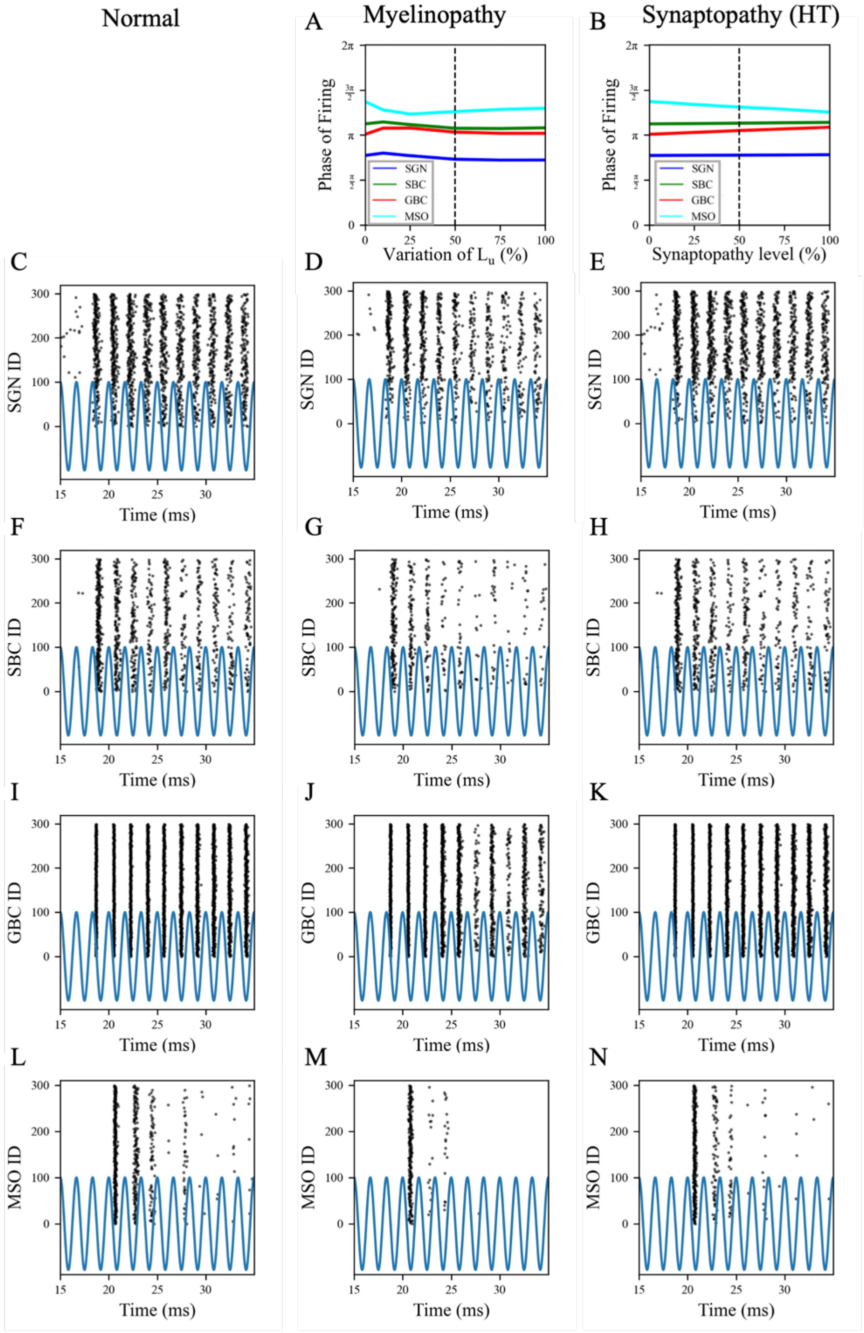

Supplementary Figure 5. Average phases of spikes of all cell types in MSO circuits do not change with higher myelinopathy or synaptopathy levels in response to 600 Hz sound stimulus. Average phases of firing, relative to the sound wave, of cell populations (SGNs, SBCs, GBCs, MSOs) with respect to 600 Hz sound stimulus in case of various (A) myelinopathy and (B) HT synaptopathy levels, where dashed lines in both panels indicate the level of myelinopathy/synaptopathy of the raster plots below (D, G, J, M for myelinopathy and E, H, K, N for synaptopathy). The raster plots of 300 SGNs (C, D and E), 300 SBCs (F, G, H), 300 GBCs (I, J, K) and 300 MSOs (L, M and N) of the control (C, F, I and L), i.e., 0% L_u variation or 0% synaptopathy, 50% L_u variation (D, G, J and M) and 50% synaptopathy (E, H, K and N), with 200 Hz sound waves superimposed on the raster plots for phase comparison.
